# Supplementary material for: Comprehensive Annotation and Functional Exploration of MicroRNAs in Lettuce
Source: Front Plant Sci. 2021 Dec 24;12:781836. doi: 10.3389/fpls.2021.781836 (PMC8739914; doi:10.3389/fpls.2021.781836)
Supplement: Supplementary file 3 [file Data_Sheet_9.ZIP › GSM2230751.plot/Lsa-miRN69_Lsat_1_v5_gn_6_78761.1_563_TPlot.pdf]

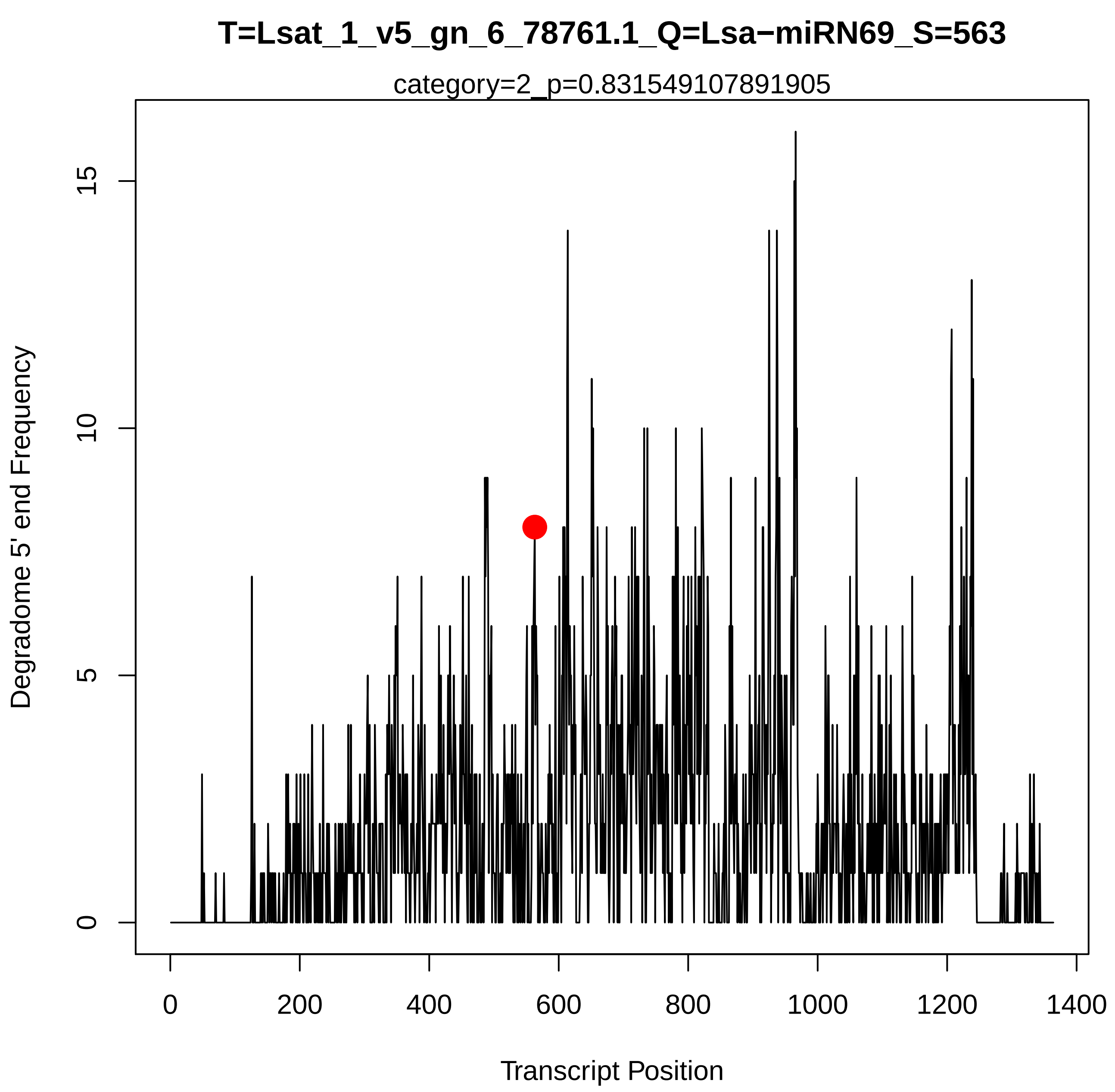

T=Lsat_1_v5_gn_6_78761.1_Q=Lsa−miRN69_S=563
category=2_p=0.831549107891905
Degradome 5' end Frequency
0
5
10
15
0
200
400
600
800
1000
1200
1400
Transcript Position
